# Iris ultrastructure in patients with synechiae as revealed by *in vivo* laser scanning confocal microscopy: In vivo iris ultrastructure in patients with Synechiae by Laser Scanning Confocal Microscopy

**DOI:** 10.1186/s12886-016-0224-2

**Published:** 2016-04-26

**Authors:** Ming Li, Hongbo Cheng, Ping Guo, Chun Zhang, Song Tang, Shusheng Wang

**Affiliations:** Key laboratory of ophthalmology, Shenzhen Eye Hospital, Ji-nan University, Shenzhen, 518000 P. R. China; Department of Cell and Molecular Biology, Tulane University, 2000 Percival Stern Hall, 6400 Freret Street, New Orleans, LA 70118 USA; Department of Ophthalmology, Tulane University, 1430 Tulane Avenue, SL-69, New Orleans, LA 70112 USA

**Keywords:** Iris, In vivo, Ultrastructure, Laser scanning confocal microscopy

## Abstract

**Background:**

Iris plays important roles in ocular physiology and disease pathogenesis. Currently it is technically challenging to noninvasively examine the human iris ultrastructure in vivo. The purpose of the current study is to reveal human iris ultrastructure in patients with synechiae by using noninvasive in vivo laser scanning confocal microscopy (LSCM).

**Methods:**

The ultrastructure of iris in thirty one patients, each with synechiae but transparent cornea, was examined by in vivo LSCM.

**Results:**

Five characteristic iris ultrastructures was revealed in patients with synechiae by in vivo LSCM, which include: 1. tree trunk-like structure; 2. tree branch/bush-like structure; 3. Fruit-like structure; 4. Epithelioid-like structure; 5. deep structure. Pigment granules can be observed as a loose structure on the top of the arborization structure. In iris-associated diseases with Tyndall’s Phenomenon and keratic precipitates, the pigment particles are more likely to fall off from the arborization structure.

**Conclusions:**

The ultrastructure of iris in patients with synechiae has been visualized using in vivo LSCM. Five iris ultrastructures can be clearly observed, with some of the structures maybe disease-associated. The fall-off of the pigment particles may cause the Tyndall’s Phenomenon positive. In vivo LSCM provides a non-invasive approach to observe the human iris ultrastructure under certain eye disease conditions, which sets up a foundation to visualize certain iris-associated diseases in the future.

**Electronic supplementary material:**

The online version of this article (doi:10.1186/s12886-016-0224-2) contains supplementary material, which is available to authorized users.

## Background

The human iris is a pigmented membranous structure located between the cornea and the lens. It has important physiological functions, including pupil size adjustment and participation in the formation of the eye darkroom structure. It also plays an important role the pathogenesis of many ocular diseases, such as uveitis and glaucoma, among others [1–4]. By histology, based on the sections from embedded pathological samples, the iris can be divided into four-layers, including the anterior limiting layer, the iris stroma and the pupil sphincter, anterior pigment myoepithelium and pupil dilatator muscle, as well as the posterior pigment epithelium. In order to test the involvement of iris in ocular disease, it would be ideal to develop a noninvasive method to examine the human iris ultrastructure.

In vivo laser scanning confocal microscopy (LSCM) has been used to visualize the layers of corneal structures at the cellular level. It has been applied clinically to reveal characteristic features in many keratopathies [5–13]. Compared to the conventional imaging methods, in vivo LSCM can achieve superior vertical and lateral resolutions and provide outstanding image contrast, which allow for rapid real-time in vivo examination of corneal ultrastructures. However, under normal conditions, the distance of the iris from the cornea prevents the visualization of iris ultrastructure using in vivo LSCM, even in the peripheral/limbal region [2–5]. Some researchers have tried to optimize in vivo LSCM imaging by changing the camera lens and increasing its working distance, but the quality of images was still subpar [14].

To understand the ultrastructure of the iris in vivo and study its relationship to iris-associated diseases, an approach have been devised to visualize iris ultrastructure using in vivo LSCM in 31 special patients who had anterior synechiae (iris-cornea contact) due to various reasons. Characteristic ultrastructures of human synechiae iris were observed through the transparent corneas of these patients. These data represent high quality noninvasive imaging of synechiae iris ultrastructures. Moreover, some of the features may be associated with the disease conditions.

## Methods

### Patients

The study was conducted in compliance with informed consent regulations and the Declaration of Helsinki. The study protocol was approved by internal review board (IRB) of Shenzhen Eye Hospital. Informed consent was obtained from the patients for the study. Eyes from 31 patients in Shenzhen Eye Hospital (Shenzhen city, P. R. China) who had synechiae but transparent cornea were examined during the period from March 2008 to March 2014. These includes14 women and17 men with an average age of 34.8 ± 5.6 years old. The causes of contact between the iris and the cornea in the patients include: eight cases of post-traumatic iridocorneal adhesions, five cases of uveitis, seven cases of iridocorneal endothelial (ICE) syndrome, three cases of angle-closure glaucoma, four cases of corneal transplantation, four cases of post-eye surgery with contacting of the iris and the cornea.

All patients were first examined and diagnosed by an ophthalmologist specialized in studying iris and cornea contact. Data collection included medical and ophthalmological history, age, age at diagnosis, gender, detailed slit-lamp examination and in vivo LSCM imaging. At the time of in vivo LSCM examination, the eye conditions were stable. All individual patients were informed of the aims of recording these data, including in vivo LSCM imaging.

### In vivo laser scanning confocal microscopy

The area with contacting of the iris and the cornea was examined by in vivo LSCM with magnification up to X800 (HRT II Rostock Cornea Module, diode laser 670 nm, Heidelberg Engineering GmbH, Germany). Images consist of 384 X 384 pixels covering an area of 400 X 400 mm with a transverse optical resolution of approximately 1 mm/pixel and an acquisition time of 0.024 s (Heidelberg Engineering).

Before microscopy examination, a drop of topical anesthetic (0.5 % Alcaine, Alcon Corp, Fort Worth, Texas USA) was applied to the lower conjunctival sac of the patients. A sterile Tomocap (Heidelberg Engineering) was mounted over the objective of the microscope (Zeiss, Jena, Germany; 363), and a drop of Vidisic gel (0.2 % Carbomer 940; Bausch & Lomb, Germany) was used as a coupling agent between the cap and the lens objective.

The patient’s head was positioned in the headstock with the eyes steadily gazed at the fixation tool. The area with contacting of the iris and the cornea was examined and images were recorded at one point along the z-axis as single scans or in the movie motion mode. Each eye was examined for less than 5 min. To standardize measurements, all images were subsequently randomized and encoded by a single independent observer.

## Results

By examining patients with iris-cornea contact, characteristic ultrastructures of human synechiae iris were detected under in vivo LSCM. The ultrastructure of human synechiae iris in vivo under the laser scanning confocal microscope resembles a tree-like structure that branches from the surface of the iris that contacts the cornea to the deeper interior. The structure gradually branches from tree trunk-like structure to tree branch or bush-like structure, then to fruit-like structure, and finally to epithelioid-like structure and deep structure. A lot of pigment clumps were observed to attach to different structures. Details of the structures are described below.

1. Tree trunk-like structure (Fig. 1a-c). This structure was observed on the surface of iris that contacts the cornea. It is a support structure with very thick arrangement, similar to a tree trunk. It showed moderate to high light reflection with no cellular structure observed under in vivo LSCM. Scattered highly light-reflective pigment clumps could be observed near the tree trunks.

**Fig. 1:**
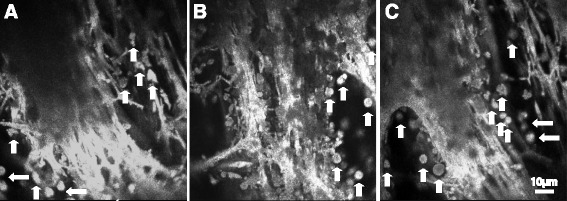
Tree trunk-like structure structures (**a**-**c**) in the human iris with synechiae by in vivo LSCM. Note the support structure with very thick arrangement, similar to the trunk with moderate to high reflection. On the left side and below, finer tree branch-like structure is visible with few scattered pigment clumps. Scale bar equals to 10 μm. Arrows show the pigment granules
2. Tree branch/bush-like structure (Fig. 2a-c). Tree branch-like structure was observed under the surface of the iris by in vivo LSCM. It has a tree branch-like or fine arborization shape. Pigment agglomerates could either be clearly seen (Fig. 2a) or not seen (Fig. 2b). Tree bush-like structure was observed from the branching of the tree branch-like structure (Fig. 2c). This structure can be associated with or without fruit-like pigment agglomerates.

**Fig. 2:**
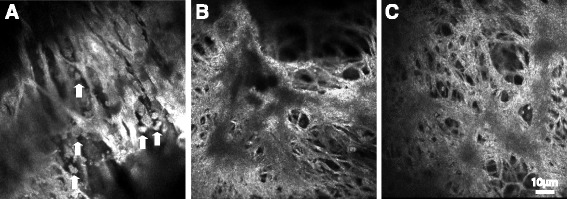
Tree branch-like structures. **a** Tree branch-like structure with fine dendritic structure with clear pigment agglomerates. **b** Tree branch-like structure without pigment agglomerates; **c** Tree bush-like structure branching from the tree branch-like structure. Scale bar equals to 10 μm. Arrows show the pigment granules
3. Fruit-like structure (Fig. 3a-b). This structure has the fruit-like pigment agglomerates and was observed at the top of the tree bush-like structure.

**Fig. 3:**
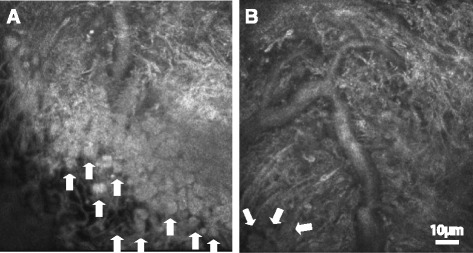
**a**-**b** Fruit-like structures showing pigment agglomerates in the tree bush-like structure. Scale bar equals to 10 μm. Arrows show the pigment granules
4. Epithelioid-like structure (Fig. 4a-b). In this structure, the epithelioid-like cells are arranged in sheets. The cell borders were highly light-reflective, while the structures inside the cells were low to medium light-reflective under in vivo LSCM. Little pigment granules could be observed at the top of epithelioid-like cells.

**Fig. 4:**
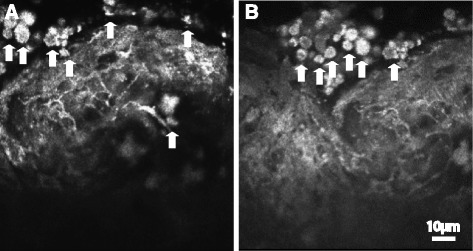
**a**-**b** Epithelioid-like structure that epithelioid cells arranged in sheets. Pigment granules can be seen at the right top of epithelioid cells. Scale bar equals to 10 μm. Arrows show the pigment granules
5. Deep structures (Fig. 5a-b). These structures were observed at the deep interior of iris by in vivo LSCM. They could be either coarse or lobular. Due to the limited penetration of the laser, so the structures were more blurred.

**Fig. 5:**
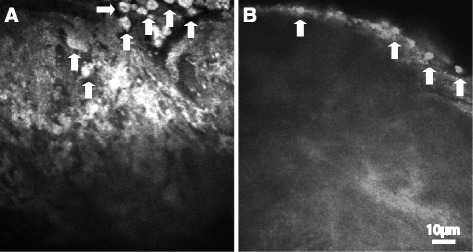
**a**-**b** Deep structure that is coarse or lobulating. Scale bar equals to 10 μm. Arrows show the pigment granules

In sum, large number of dendritic scaffold structures can be observed on the iris surface by in vivo LSCM. They grow and extend like tree branches, tapering gradually from coarse to fine branches. With branching, the corresponding pigment clumps and particles are increasing gradually, forming clusters of fruit structure similar to hanging bracket tip. For example, there are few pigment granules located at the tree-trunk and branch-like structures in Fig. 1a-b. However, many pigment granules were seen in the epitheliod-like structure in Fig. 4. In a variety of eye diseases with pigment particles falling from iris known as Tyndall’s Phenomenon and keratic precipitates, the pigment particles are likely to fall off from the branches. This can be observed in Figs. 1, 4 and 5 (showing pigment particles falling off).

**Fig. 6:**
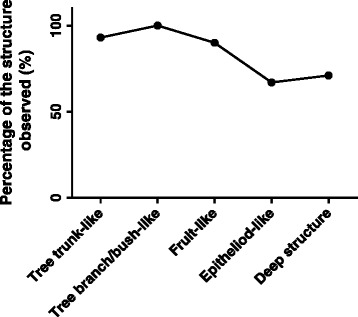
Percentage of different iris ultrastructures in 31 patients with synechiae

Statistically, the percentage of different structures were found to be different in different patients (Fig. 6 and Additional file 1: Table S1). While tree trunk-like structure, tree branch/bush-like structure and fruit like structure were found in more than 90 % of the patients with synechiae, the epitheloid-like and deep structures were observed in about 70 % of the patients. In 3 of 8 cases of post-traumatic iridocorneal adhesions and 2 of 7 cases of iridocorneal endothelial syndrome, the epitheliod-like and deep structures were not observed. Therefore, the 5 types of structures were not necessarily observed in every patient, which may due to the technical limitation of the in vivo LSCM under the disease conditions.

## Discussion

The iris is a disc-shaped membrane structure in the front-most portion of the uveal. The center of the disc is called pupil, which can adjust the amount of light entering the eye. Iris plays an important role in many eye diseases [15, 16]. Clinically, to observe iris, the most common method is to use slit lamp microscope. New technologies, such as UBM (ultrasound biomicroscopy), OCT (optical coherence tomography), are also used in clinical observation of the iris nowadays [17]. However, the resolution of these technologies is limited, and is not suitable for observing iris ultrastructures.

In vivo LSCM can magnify images for up to 800 times, representing an excellent tool to visualize iris ultrastructure. Due to the working distance, the iris structure cannot be observed by in vivo LSCM under normal circumstances since the iris is away from the cornea [18, 19]. Even when the Nikon lens of in vivo LSCM was changed to increase its working distance, the acquired iris image was still not clear [14].

We took advantage of the patients with iris-cornea contact which were caused by trauma, eye surgery and diseases. By adjusting the focal length of in vivo LSCM, as well as the laser through the cornea into the iris contacting, we successfully obtained high resolution human synechiae iris ultrastructure [20–22].

By in vivo LSCM, The ultrastructure of iris can be divided into the following five characteristic types: (1) tree trunk-like structure (Fig. 1a); (2) tree branch/bush-like structure (Fig. 1b-d); (3) Fruit-like structure (Fig. 1e); (4) epithelioid-like structure (Fig. 1f); and (5) deep structure (Fig. 1g-h). These structures are different from and cannot substitute what described from the histopathological structures of the iris after conventional sample dehydration, embedding and sectioning, but they represent the real-time and live-cell iris structure in a non-invasive condition. Of note, it is currently difficult to directly correlate the five characteristic structures in our non-invasive in vivo studies with the sections from the histopathological studies.

We found that the support structure of the iris branches gradually from coarse to fine. Based on the size of the support structure, the stent surface of iris can be divided into three classes of structures: tree trunk-, tree branch/bush- and fruit-like structure. Epitheliod-like structure, which we define as the class 4 structure, is formed on the epithelial surface at the base of the stent-like structure. Even with our special samples, due to the existence of numerous iris pigment particles and the support structure, as well as the limited penetration of the laser, the ultrastructure of the iris deep imaging is still not clear, which represents a limitation of this technology [8]. The shallower in position of the iris, the clearer is the image. On the contrary, the deeper in position, the blurrier the image. We define these deep structures as class 5 structure. We also observed a lot of pigments in the iris covering the stent surface, and the pigment particles stand up at the tip. We found in some cases the pigment particles fall off from the surface, likely resembling disease conditions with pigment particles falling from iris known as Tyndall’s Phenomenon and keratic precipitates.

Taken together, in this study, the ultrastructure of iris contacting with cornea has been visualized using in vivo LSCM. Especially, among the structures, the scaffold structure of the iris surface and pigment clumps can be clearly observed. Since what we have observed are the in vivo ultrastructures of the iris under the conditions of iris-cornea contact, we expect that some of the structures are disease associated, while some represent the normal iris structure. Indeed, the fall-off of pigment particles observed by our in vivo assay may shed light on the mechanism underlying the Tyndall’s phenomenon in iris-associated diseases. Since we only have 31 cases in this study, it is still too early to correlate the specific changes in the in vivo ultrastructure to specific iris-associated diseases.

## Conclusions

Using in vivo LSCM, the ultrastructure of iris in patients with synechiae has been visualized. Five iris ultrastructures can be clearly observed, with some of the structures maybe disease-associated. Our study provides a non-invasive approach to visualize the ultrastructure of the iris, which sets up a foundation to visualize certain iris-associated diseases in the future.

## Ethics

The study protocol was approved by internal review board (IRB) of Shenzhen Eye Hospital.

## Consent to publish

Informed consent was obtained from the patients for the study. Since the study contains no individual persons’ data, there is no requirement for consent to publish.

## Availability of data and materials

All the data supporting your findings is contained within the manuscript.

## Additional file

Additional file 1: Table S1. Detailed structures observed in individual patients with synechaie. (DOCX 13 kb)

## Abbreviations

LSCM: Laser scanning confocal microscopy
UBM: Ultrasound biomicroscopy
OCT: Optical coherence tomography
