# Supplementary material for: Iris ultrastructure in patients with synechiae as revealed by in vivo laser scanning confocal microscopy: In vivo iris ultrastructure in patients with Synechiae by Laser Scanning Confocal Microscopy
Source: BMC Ophthalmol. 2016 Apr 26;16:46. doi: 10.1186/s12886-016-0224-2 (PMC4847242; doi:10.1186/s12886-016-0224-2)
Supplement: Additional file 1: Table S1. — Detailed structures observed in individual patients with synechaie. (DOCX 13 kb) [file 12886_2016_224_MOESM1_ESM.docx]

**Supplemental Table 1. The structures observed in 31 patients.**

| Patient Number | Groups | Structures (+: observed; -: not observed) | | | | |
| --- | --- | --- | --- | --- | --- | --- |
|  |  | Tree trunk-like | Tree branch /bush-like | Fruit structure | Epithelioid-like | Deep structures |
| 1 | post-traumatic iridocorneal adhesions | + | + | + | + | + |
| 2 | uveitis | + | + | + | + | + |
| 3 | post-traumatic iridocorneal adhesions | + | + | + | + | + |
| 4 | uveitis | + | + | + | + | + |
| 5 | iridocorneal endothelial syndrome | + | + | + | + | + |
| 6 | post-traumatic iridocorneal adhesions | + | + | + | - | - |
| 7 | angle-closure glaucoma | + | + | + | + | + |
| 8 | corneal transplantation | + | + | + | + | + |
| 9 | post-traumatic iridocorneal adhesions | + | + | + | + | - |
| 10 | post-eye surgery with contacting of the iris and the cornea | + | + | + | + | + |
| 11 | uveitis | + | + | + | + | + |
| 12 | iridocorneal endothelial syndrome | + | + | + | - | - |
| 13 | corneal transplantation | + | + | + | + | + |
| 14 | post-traumatic iridocorneal adhesions | + | + | + | - | - |
| 15 | iridocorneal endothelial syndrome | + | + | + | - | - |
| 16 | uveitis | + | + | + | - | - |
| 17 | post-eye surgery with contacting of the iris and the cornea | + | + | + | - | - |
| 18 | post-traumatic iridocorneal adhesions | + | + | + | + | + |
| 19 | angle-closure glaucoma | + | + | + | + | + |
| 20 | post-eye surgery with contacting of the iris and the cornea | + | + | + | + | + |
| 21 | uveitis | + | + | - | - | - |
| 22 | iridocorneal endothelial syndrome | + | + | + | + | + |
| 23 | corneal transplantation | + | + | + | + | + |
| 24 | post-traumatic iridocorneal adhesions | - | + | - | - | - |
| 25 | post-eye surgery with contacting of the iris and the cornea | + | + | + | + | + |
| 26 | iridocorneal endothelial syndrome | + | + | + | + | + |
| 27 | iridocorneal endothelial syndrome | + | + | + | + | + |
| 28 | corneal transplantation | - | + | - | - | - |
| 29 | iridocorneal endothelial syndrome | + | + | + | + | + |
| 30 | post-traumatic iridocorneal adhesions | + | + | + | + | + |
| 31 | angle-closure glaucoma | + | + | + | + | + |
